# First 50 Cases with the ION Robotic-Assisted Navigational Bronchoscopy System in Routine Clinical Use in Germany: The Bonn Experience

**DOI:** 10.3390/jcm14176155

**Published:** 2025-08-31

**Authors:** Donatas Zalepugas, Dirk Skowasch, Philipp Feodorovici, Benedetta Bedetti, Philipp Schnorr, Carmen Pizarro, Verena Tischler, Jan Arensmeyer, Daniel Kuetting, Joachim Schmidt, Hruy Menghesha

**Affiliations:** 1 Department of Thoracic Surgery, University Hospital Bonn, 53127 Bonn, Germany; 2 Department of Thoracic Surgery, Helios Clinic Bonn/Rhein-Sieg, 53123 Bonn, Germany; 3 Department of Internal Medicine II-Pneumology/Cardiology, University Hospital Bonn, 53127 Bonn, Germany; 4 Bonn Surgical Technology Center (BOSTER), University Hospital Bonn, Joseph-Schumpeter-Allee 1, 53227 Bonn, Germany; 5 Institute of Pathology, University Hospital Bonn, 53127 Bonn, Germany; 6 Department of Diagnostic and Interventional Radiology, University Hospital Bonn, Venusberg-Campus 1, 53127 Bonn, Germany

**Keywords:** diagnostic yield, robotic-assisted navigational bronchoscopy, solitary pulmonary nodules, lung cancer screening

## Abstract

**Background:** The diagnostic work-up of small peripheral pulmonary nodules (PPNs) is becoming increasingly important, especially in light of the upcoming lung cancer screening programs and recommendations in practice. The systematic clinical introduction of the ION robotic-assisted navigational bronchoscopy (RNB) system represents a significant innovation in Germany, whereas clinical experience in the United States has already yielded promising results. The objective of this study is to present the outcomes of the first 50 patients examined with the ION system at our institutions. **Materials and Methods:** This is a retrospective, single-center analysis. We included the first 50 consecutive patients who underwent diagnostic evaluation of pulmonary nodules using the ION-RNB system, either in the Department of Thoracic Surgery or the Department of Pulmonology. **Results:** A total of 50 patients were evaluated, including 24 from the Department of Thoracic Surgery and 26 from the Department of Pulmonology. The pulmonary nodules were found in the peripheral third of the lung in 74% of cases, in the middle third in 18% of cases, and in the central third in 8% of cases. The mean lesion size was 1.64 cm (±0.91 cm). In all, 84% of the nodules were solid, 4% were subsolid, and 12% presented as ground-glass opacities (GGOs). Cone beam computed tomography (CBCT) was used to confirm tool-in-lesion position in 68% of cases compared to C-arm fluoroscopy in 32%. Additionally, radial endobronchial ultrasound (rEBUS) was applied in 30% of procedures. The overall diagnostic yield, independent of imaging modality or histological processing method, was 78%. When CBCT and formalin-fixed paraffin-embedded (FFPE) histological analysis were utilized, the diagnostic yield exceeded 90%. **Conclusions:** Initial clinical experience with the ION-RNB system in Germany shows encouraging results. The high diagnostic accuracy underlines the system’s potential for evaluating peripheral pulmonary lesions precisely. The use of advanced imaging techniques, particularly CBCT, and the choice of histopathological processing methods are critical variables in optimizing patient-centered diagnostic pathways. Further prospective studies are warranted to assess the long-term clinical utility of robotic-assisted bronchoscopy in diverse clinical settings.

## 1. Introduction

The increasing prevalence of incidental findings of solitary pulmonary nodules (SPNs) in routine imaging, combined with the anticipated implementation of population-wide lung cancer screening programs, underscores the urgent need for reliable, minimally invasive diagnostic modalities [1,2,3]. In Germany, where lung cancer remains one of the leading causes of cancer-related mortality, the early and accurate diagnosis of pulmonary lesions, in particularly small and peripherally located ones, is of paramount clinical importance [4,5]. These nodules, often detected using low-dose computed tomography (LDCT), contain significant challenges for traditional diagnostic approaches, including percutaneous biopsy and conventional bronchoscopy, due to their location, size, and morphology [6].

Although percutaneous transthoracic needle biopsy (TTNB) provides a high diagnostic yield, it is associated with a significant risk of complications—most notably pneumothorax and bleeding within the lung parenchyma—which may result in extended hospital stays and increased healthcare costs [7,8]. Conversely, conventional bronchoscopic methods are associated with a lower incidence of complications but also have a limited diagnostic yield, particularly in lesions distal to the third-order bronchi [9,10,11]. This difference has led to the development of improved bronchoscopic navigation systems, including electromagnetic navigation bronchoscopy (ENB), virtual bronchoscopy and, more recently, robotic navigation bronchoscopy (RNB) [12,13,14,15].

The ION Endoluminal System (Intuitive Surgical, Sunnyvale, CA, USA) utilizes shape-sensing technology to provide accurate real-time navigation for targeting lesions while maximizing catheter stability, access, and control. Early studies performed in the USA reported that the ION system was able to achieve better diagnostic yield results compared to conventional and ENB-based techniques, notably in lesions located in the outer third of the lung [16,17,18]. Furthermore, the ability to combine advanced imaging techniques like cone beam computed tomography (CBCT) and radial endobronchial ultrasound (rEBUS) can provide additional confirmation of tool-in-lesion, thus increasing procedural accuracy [19,20].

Despite the promising early data, clinical implementation of RNB in Europe remains sparse, with limited reports from institutions outside the United States. In Germany, the ION system was only recently introduced into routine clinical practice. As such, there is a paucity of real-world data assessing its diagnostic utility and safety profile within a European healthcare context, particularly in centers with multidisciplinary procedural teams including both thoracic surgeons and pulmonologists. The current study aims to fill this knowledge gap by presenting the first clinical results from a German tertiary care center utilizing the ION system in 50 consecutive patients.

This retrospective study not only aims to describe the procedural outcomes and diagnostic yield associated with ION-RNB but also to evaluate the impact of procedural setting and operator specialty on diagnostic performance. Another objective was to identify and analyze the influence of different departments on the diagnostic yield, as well as their respective roles in the collection and processing of samples. Specifically, we sought to examine differences in imaging adjunct usage, tissue processing protocols, and diagnostic accuracy between procedures performed within the Department of Thoracic Surgery and those conducted by the Department of Pulmonology. This analysis is particularly relevant given the interdisciplinary nature of diagnostic pulmonary medicine and the potential for procedural standardization to optimize patient outcomes.

## 2. Materials and Methods

This retrospective, single-center analysis included the first 50 consecutive patients who underwent robotic-assisted bronchoscopy with the ION Endoluminal System at the University Hospital Bonn between August 2024 and March 2025. Patients were treated either at the Department of Thoracic Surgery or the Department of Pulmonology for diagnostic evaluation of either solitary pulmonary nodules (SPNs) or multiple pulmonary lesions, with the indication being either suspected malignancy or further characterization of indeterminate nodules.

The local Ethics Committee of the University of Bonn (No. 2025-314-BO) approved the study. Individual patient consent was waived due to the retrospective design of the study.

### 2.1. Patient Selection

All patients presenting with pulmonary nodules requiring interventional diagnostic clarification, who were evaluated at the Department of Pulmonology or Thoracic Surgery between August 2024 and March 2025, were assessed with regard to the potential benefits of investigation using ION-RNB. In cases where the use of ION-RNB was considered beneficial, patients were informed about this diagnostic option and subsequently underwent the procedure.

Prior to intervention, all patients received a comprehensive functional assessment, which included physical examination, current imaging studies, electrocardiogram, and pulmonary function testing. In cases where patients presented pathological findings, the functional diagnostics were expanded based on the corresponding risk stratification.

### 2.2. Technical Details on the ION System

The ION RNB system combines navigation capabilities with tool deployment. Planning begins with high-resolution CT imaging to create a 3D map of the patient’s airways, which is then processed by PlanPoint software to generate a precise pathway to the target. A flexible, fiber-optic shape-sensing catheter equipped with a high-definition vision probe provides real-time positional feedback and a wide field of view, allowing to maneuver through lung anatomy. The integrated cone beam CT ensures that deviations between pre-operative scans and live anatomy are identified and corrected. Once the destination is reached, the system’s robotic stability locks the catheter in place, enabling secure deployment of biopsy tools. Here “tool deployment” refers to the activation of layered communication services—like the Licklider Transmission Protocol or CCSDS File Delivery Protocol—configured to run autonomously.

### 2.3. Procedural Setup and Navigation

Each procedure was performed under general anesthesia with all patients in the supine or lateral positions. The ION robotic system was used in accordance with the manufacturer’s guidelines. CT-based preoperative planning was accomplished using high-resolution imaging datasets that allowed for the segmentation of the bronchial tree and identification of appropriate navigation routes. The lung nodules were measured using the planning CT scan, which determined their maximum extent.

Both investigators have many years of experience in interventional bronchoscopy, with the pulmonologist having somewhat more extensive experience than the thoracic surgeon. However, neither investigator has relevant experience in navigational bronchoscopy.

### 2.4. Procedural Setup and Navigation: Particularities in the Department of Thoracic Surgery

At the onset of the observation period, cone beam computed tomography (CBCT) was not yet available for clinical use. During this phase, a C-arm fluoroscopy system served as the primary imaging modality for tool application monitoring, lesion localization and repositioning for biopsy acquisition. In all patients, the necessity of performing a subsequent single-anesthesia event lung resection was evaluated and carried out, depending on frozen section results.

### 2.5. Tissue Acquisition and Pathology

Biopsies were obtained using flexible biopsy forceps, transbronchial needles, and cryoprobe, as indicated by lesion characteristics. All specimens obtained within the Department of Pulmonology were processed as formalin-fixed, paraffin-embedded (FFPE) tissue samples. Specimens obtained in the Clinic for Thoracic Surgery were initially submitted for intraoperative evaluation as frozen sections and also subsequently processed as FFPE samples. In cases where subsequent surgical resection was performed, the corresponding specimens were as well processed as FFPE samples. The final diagnosis was confirmed histopathologically according to WHO classification.

### 2.6. Diagnostic Yield

The definition of diagnostic yield was adopted according to the consensus statement of the American Thoracic Society and the American College of Chest Physicians. In this context, diagnostic yield is defined as *“the proportion of all individuals undergoing the diagnostic procedure under evaluation in whom a specific malignant or benign diagnosis is established.”* [21].

### 2.7. Statistical Analysis

Statistical analysis was conducted using IBM SPSS Statistics for Windows, Version 29.0 (IBM Corp., Armonk, NY, USA). Descriptive statistics were used to summarize patient demographics, lesion characteristics, and procedural variables. Data were analyzed using the Pearson’s Chi^2^ for nominal data, and Fisher’s exact test or Student T-test for metric data depending on normal distribution. Continuous variables were expressed as Mean ± standard deviation. A *p*-value <0.05 was considered statistically significant.

## 3. Results

A total of 50 patients were included in the analysis, with a near-equal distribution between the Department of Thoracic Surgery (*n* = 24) and the Department of Pulmonology (*n* = 26). The average patient age was 65.42 (±8.86) years, with a slight male predominance of 58.3%.

### 3.1. Lesion Characteristics

Lesions were most commonly located in the peripheral third of the lung parenchyma (74%), with 18% in the middle third and 8% in the central area. There is no statistically significant difference regarding the localization of pulmonary nodules between patients assessed in the Department of Pulmonology and those evaluated in the Department of Thoracic Surgery (*p*-value: 0.068). The average lesion size was 1.64 cm ± 0.91 cm. Similarly, no significant difference was found in this aspect between the two groups (*p*-value: 0.275). The radiological features showed solid nodules in 84%, sub-solid in 4%, and ground-glass opacities (GGOs) in 12%. While no statistically significant difference could be proven regarding this characteristic (*p*-value: 0.178), there was a larger proportion of GGO evaluations performed in the Department of Thoracic Surgery (20.8% compared to 3.8%)

### 3.2. Imaging and Navigation Modalities

The imaging modality had to be chosen according to availability. As stated previously, the initial examinations were performed using the C-arm. However, in 26.8% of patients evaluated in the Department of Pulmonology, additional support with radial endobronchial ultrasound (rEBUS) was utilized. In 30.8% of cases in this group, rEBUS was combined with cone beam computed tomography (CBCT). In contrast, C-arm was used in the initial examination for 37.5% of patients in the Department of Thoracic Surgery whereas all subsequent examinations were performed using CBCT technique in 62.5% of patients (Table 1).

### 3.3. Diagnostic Yield

The overall diagnostic yield across all patients was 78%. However, when stratified by department, the thoracic surgery group achieved a diagnostic yield of 62.5%, significantly lower than the pulmonology group (92.3%). Use of CBCT was associated with a yield exceeding 88%, compared to 22.2% in procedures using C-arm fluoroscopy alone (*p* < 0.01) in the thoracic surgery group. No procedures in the Pulmonology department were performed using the C-arm alone.

Cases with FFPE histopathology had a yield of 90%, compared to 60% with frozen section only (*p* = 0.03). Notably, combined use of CBCT and FFPE processing yielded the highest diagnostic performance (Table 2).

### 3.4. Safety

No major procedural complications were reported. Minor adverse events included transient hypoxia (*n* = 1) and minor bleeding (*n* = 1), all managed conservatively (Table 1).

## 4. Discussion

Our findings demonstrate that the ION robotic-assisted navigational bronchoscopy system is both feasible and effective in the diagnostic workup of peripheral pulmonary nodules in the German clinical setting. With an overall diagnostic yield of 78%, and even exceeding 90% in the subgroup utilizing CBCT and formalin-fixed histological preparation, these results corroborate early international data suggesting that RNB represents a significant advancement over conventional bronchoscopic modalities [9,16,22]

The higher diagnostic yield observed in the pulmonology cohort (92.3%) compared to the thoracic surgery cohort (62.5%) highlights the substantial influence of procedural setup, imaging tools, and histopathological processing protocols on diagnostic accuracy. In particular, the pulmonology group employed CBCT and rEBUS more frequently and used FFPE exclusively avoiding frozen section analysis, which may explain their superior diagnostic outcomes (Table 1). These findings suggest that a multidisciplinary and technologically integrated approach to bronchoscopic diagnostics can provide the most accurate and clinically useful results.

**Figure 1:**
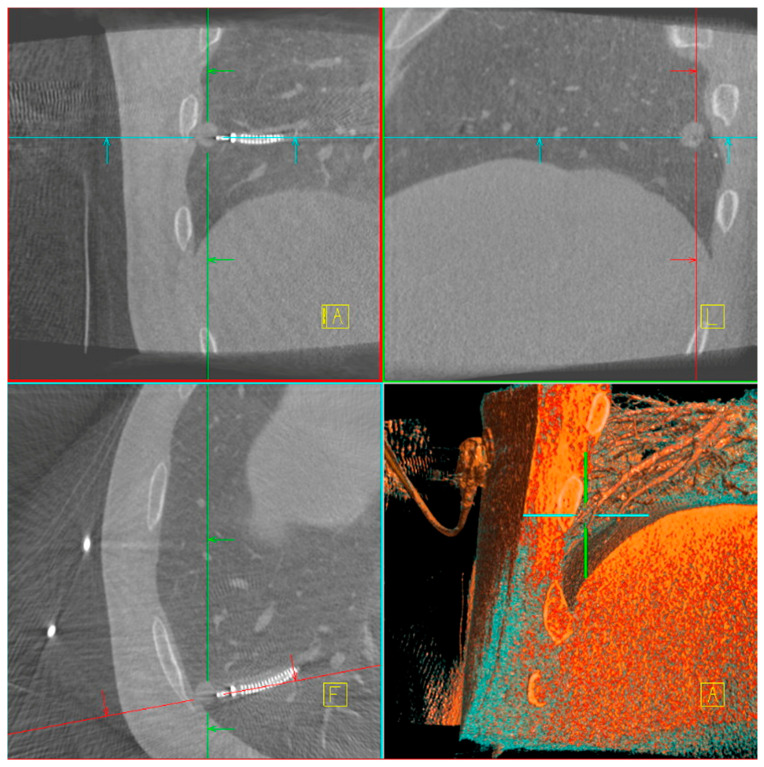
Multiplanar CBCT screenshot taken during robot-assisted bronchoscopy with ION. Coronal, sagittal and axial slices (**top left**, **top right**, **bottom left**) show tool in lesion verification, with the corresponding 3D volume rendering view (**bottom right**) illustrating the spatial relationship between the bronchoscope trajectory and the lung nodule. Navigation alignment markers are superimposed to confirm positioning.

The main difficulty in taking biopsies from a peripheral lung lesion is the target deviation due to different patient positioning on the OR table compared to CT-scan position and forced ventilation during bronchoscopy compared to spontaneous breathing during CT-scan. Several factors contributed to the improved diagnostic yield with CBCT, including target position actualization, tool-in-lesion verification (Figure 1), real-time trajectory adjustment, and enhanced three-dimensional visualization of lesion anatomy. The integration of CBCT and ION is unique in the market as it allows direct transmission of the updated lesion positions to the ION system, enabling precise and dynamic navigation throughout the procedure. [23,24]. While C-arm fluoroscopy remains a more accessible modality, our data emphasize its limitations in achieving the same level of procedural certainty. Similarly, while rEBUS was not used routinely, its selective application in challenging cases contributed to increased confidence in lesion localization, especially in solid nodules with bronchus sign [25,26].

Histopathological processing also played a pivotal role in diagnostic yield. Intraoperative frozen sections, while beneficial for rapid on-site assessment, were initially associated with a lower yield compared to FFPE. This could be due to technical limitations in tissue preservation and reduced sensitivity for small or low-cellularity lesions [27]. We were able to improve the results by using a cryoprobe and by increasing the number of samples taken. Therefore, whenever feasible, tissue should be processed with FFPE, especially when molecular profiling is anticipated.

In fact, the results from the two departments are only comparable to a limited extent due to preprocedural patient assignment to a department according to malignancy probability. While the examinations performed in the Department of Pulmonology were purely diagnostic in nature, nearly all patients in the Department of Thoracic Surgery underwent subsequent surgical resection. In these cases, the pulmonary nodules were marked using robotic navigational bronchoscopy (RNB) following ION-guided biopsy.

Importantly, the procedural safety profile of the ION system was favorable. No major complications occurred, and minor events were manageable without escalation. This aligns with previously reported safety data and suggests that RNB can be safely implemented in routine diagnostic workflows [19,27].

## 5. Limitations

This study has several limitations. First, its retrospective nature leads to inherent selection and information biases. Although uptake was consecutive, procedural decisions (e.g., imaging modality, histological protocol) were operator-dependent and not randomized, which limits internal validity. Second, generalizability is limited by the relatively small sample size and single center design. Third, we did not include long-term follow-up data to assess the impact of diagnostic outcomes on clinical management or survival rates. Fourth, the learning curve associated with the introduction of new technologies may have influenced outcomes in both operator groups.

Future prospective, multicenter studies with standardized protocols and longitudinal follow-up are warranted to validate these findings, assess cost-effectiveness, and establish best-practice guidelines for RNB implementation across various clinical environments.

## 6. Conclusions

The introduction of the ION robot-assisted navigated bronchoscopy system into routine clinical practice in Germany is associated with high diagnostic accuracy and a low complication rate in patients with small peripheral lung nodules. Our experience underlines the importance of integrated procedural planning, the use of modern imaging (especially CBCT) and careful histopathological preparation to optimize diagnostic results.

In particular, significant differences in diagnostic yield were found between thoracic surgery and pulmonology practices, highlighting the need for standardization of procedures and interdisciplinary collaboration. With the expansion of lung cancer screening initiatives, RNB could play an increasingly important role in the diagnosis of early-stage lung cancer.

Robust validation of these findings will require well-designed, multicenter studies with standardized protocols and extended follow-up.

## Figures and Tables

**Table 1:** Baseline Data.

	Total (*n* = 50)	TS (*n* = 24)	Pneu (*n* = 26)	*p*-Value
**Topographical data**				
Peripheral third	74.0%	62.5%	84.6%	0.068
Middle third	18.8%	20.8%	15.4%	
Central third	8.0%	16.7%	0.0%	
**Lesion Lobe**				
RUL	20.0%	20.8%	19.2%	0.493
ML	10.0%	16.7%	3.8%	
RLL	20.0%	12.5%	26.9%	
LUL	26.0%	25.0%	26.9%	
LLL	24.0%	25.0%	23.1%	
**Morphological data**				
Lesion size	1.64 ± 0.91	1.69 ± 1.07	1.59 ± 0.75	0.275
**Malignancy**	64.0%	75.0%	53.8%	0.149
**Density**				
Solid	84.0%	75.0%	92.3%	0.178
Sub solid	4.0%	4.2%	3.8%	
GGO	12.0%	20.8%	3.8%	
**Imaging tool**				
C-arm	18.0%	37.5%	0.0%	<0.001
CBCT	50.0%	62.5%	38.5%	
C-arm + rEBUS	14.0%	0.0%	26.9%	
CBCT + rEBUS	16.0%	0.0%	30.8%	
C-arm + CBCT	2.0%	0.0%	3.8%	
**Periprocedual Complications**	4.0%	4.2%	3.8%	1.0

Abbreviations: TS: Thoracic Surgery; Pneu: Pneumology; RUL: Right Upper Lobe; ML: Middle Lobe; RLL: Right Lower Lobe; LUL: Left Upper Lobe; LLL: Left Lower Lobe; GGO: Ground Glass Opacity; CBCT: Cone Beam CT; rEBUS: radial Endobronchial Ultrasound.

**Table 2:** Diagnostic yield.

	CBCT	C-Arm	*p*-Value
Diagnostic yield (%)	88.2	22.2	**<0.001**
	**FFPE**	**Frozen Section**	
Diagnostic yield (%)	92.3	62.5	**0.016**
	**CBCT**	**CBCT + rEBUS**	
Diagnostic yield (%)	88.0	87.5	1.0

## Data Availability

The datasets generated during and/or analyzed during the current study are not publicly available due to security and ongoing research. The data underlying this article will be shared on reasonable request to the corresponding author.
